# Analysis and Comparison of Spatial–Temporal Entropy Variability of Tehran City Microclimate Based on Climate Change Scenarios

**DOI:** 10.3390/e21010013

**Published:** 2018-12-24

**Authors:** Abdolazim Ghanghermeh, Gholamreza Roshan, José A. Orosa, Ángel M. Costa

**Affiliations:** 1 Department of Geography, Golestan University, ShahidBeheshti, 49138-15759 Gorgan, Iran; 2 Department of Navigation Science and Marine Engineering, Energy and Propulsion Research Group, University of A Coruña, Paseo de Ronda 51, 15011 A Coruña, Spain

**Keywords:** global warming, climatic modeling, spatial analysis, urban sprawl, Shannon entropy

## Abstract

Urban microclimate patterns can play a great role for the allocation and management of cooling and heating energy sources, urban design and architecture, and urban heat island control. Therefore, the present study intends to investigate the variability of spatial and temporal entropy of the Effective Temperature index (ET) for the two basic periods (1971–2010) and the future (2011–2050) in Tehran to determine how the variability degree of the entropy values of the abovementioned bioclimatic would be, based on global warming and future climate change. ArcGIS software and geostatistical methods were used to show the Spatial and Temporal variations of the microclimate pattern in Tehran. However, due to global warming the temperature difference between the different areas of the study has declined, which is believed to reduce the abnormalities and more orderly between the data spatially and over time. It is observed that the lowest values of the Shannon entropy occurred in the last two decades, from 2030 to 2040, and the other in 2040–2050. Because, based on global warming, dominant areas have increased temperature, and the difference in temperature is reduced daily and the temperature difference between the zones of different areas is lower. The results of this study show a decrease in the coefficient of the Shannon entropy of effective temperature for future decades in Tehran. This can be due to the reduction of temperature differences between different regions. However, based on the urban-climate perspective, there is no positive view of this process. Because reducing the urban temperature difference means reducing the local pressure difference as well as reducing local winds. This is a factor that can effective, though limited, in the movement of stagnant urban air and reduction of thermal budget and thermal stress of the city.

## 1. Introduction

In the last years, due to a growing population leading to rapid urban development, urban heating has been observed [1,2,3,4]. High temperatures in urban environments can have an impact on health, economics, recreation, and the quality of life as a whole. It can also make problems for vulnerable people by increasing heat-stress in cities [5,6,7,8,9]. Fouillet (2006) [10] in France and Hajat (2007) [11] in the United Kingdom proved the relationship between the increase in temperature and mortality [10,11]. Another unfavorable impact of city heating is the increase in energy consumption. Shen et al. (2013) [12] indicate the need for energy consumption in the administrative units in the Hangzhou, China with 10.8 percent for every half-degree increase in temperature. In a similar study, Akbari et al. (2001) [13] earned an increase in electric power consumption in Los Angeles, USA, by 2–4 percent per degree of summer temperature rise. Increasing energy consumption in order to cool the spaces, has other adverse effects, such as increased carbon emissions and greenhouse gases [14], and air pollution [15,16]. 

Eventually, rising temperatures in cities are an important issue that affects all aspects of urban life, and that the lack of attention to them will result in irreversible effects. Causes of temperature rise can be observed in various branches. However, what is clear is that the impact of city expansion has a direct impact on this process [17]. Meanwhile, the expansion of major cities in recent decades has had a significant impact on climate change and the transformation of climate and micro-climatic conditions [18]. Tehran, as one of the world’s largest metropolises, has progressed much faster than its natural course, despite the rapid growth of its population, the area and size of the city has also been experiencing rapid growth in recent decades [19,20].

This city is about 160 times greater than the rule of Shah Tahmasb in 1524–1576 with an area of four square kilometers, compared to 1891 with 5.24 square kilometers, about 5.16 times, and five times larger compared to 1998 with 130 square kilometers. The figures are indicative of the accelerated physical growth of the city, even faster than its population growth. For instance, if the urban population of Tehran was compared in 1996, it was estimated that the growth rate was only about 7.3 times, which is very low relative to the physical growth of the city [21]. Therefore, this urban expansion has caused changes and evolutions in some of the climatic factors in Tehran [22,23,24,25,26]. One of the methods for studying the order and disorder in the occurrence of natural phenomena is the use of entropy. However, entropy is a non-parametric approach and a robust measure of variability. Moreover, entropy does not change drastically by small changes in [27].

In a bulk of studies, this indicator is used, and the applications of this indicator have also been of interest to researchers in the field of climatic and meteorological studies [28,29,30,31]. Despite the use of entropy method in international climate and meteorological studies [32,33,34], the capabilities and potentials of this index have not been significantly investigated in the field of climatology in Iran. Thus, the present study is one of the first researches in the country that seeks to fill the gap in this research area. 

Accordingly, the present study sought to investigate the variability of the temporal–spatial entropy of the ET bioclimatic index for the two basic periods from 1971 to 2010 and from 2011 to 2050 in Tehran. Therefore, this research seeks to answer the question if the effect of global warming can cause changes in the Spatial and Temporal entropy of the ET bioclimatic index in Tehran city or not?

## 2. Materials and Method

In this study, the Effective Temperature Index (ET) was used to evaluate the monitoring and advance of bioclimatic conditions in Tehran. Therefore, in order to calculate this index, two different time series data have been used. In this study, the baseline data will include a timeline from 1971 to 2010 and another simulated data for the years 2011–2050. In Section 2.2, the general circulation model of the atmosphere has been used and the modeling processes for simulating the climate data of the study period from 2011 to 2050 were fully explained.

The climatic data used to model the bioclimatic conditions include dry-bulb temperature and wet-bulb temperature. The data are available monthly for the base period. To diagnose these climatic parameters for the future, the outcomes of the Amon_GISS-E2-H-cc large scale model prediction outsourcing are to be deduced from bcsd data, the National Aeronautics and Space Administration (NASA) of the United States, with a resolution of 2.5 × 2.5 degrees. 

The climate modeling program at Goddard Institute for Space Studies (GISS) is primarily aimed at the development of coupled atmosphere-ocean models for simulating Earth’s climate system. Primary emphasis is placed on investigation of climate sensitivity—globally and regionally, including the climate system’s response to diverse forces, such as solar variability, volcanoes, anthropogenic and natural emissions of greenhouse gases and aerosols, paleo-climate changes, etc.

A major focus of GISS GCM simulations is to study the human impact on the climate as well as the effects of a changing climate on society and the environment. More details on the GISS GCM model are available at this reference [35].

The international climate modeling community has adopted four RCPs through the IPCC [36,37,38]. The scenarios range from RCP 8.5, which corresponds to a “non-climate policy” scenario translating into high severity climate change impacts, to RCP 2.6, which is a future requiring stringent climate policy to limit greenhouse gas emissions translating into low severity impacts [38]. 

Two middle scenarios, RCPs 4.5 and 6.0 were selected by the IPCC to be evenly spaced between RCPs 2.6 and 8.5. Together, these scenarios represent the range of radiative forces available in the peer-reviewed literature at the time of their development in 2007 [36,39]. In present work, we used RCPs 4.5 and 8.5.

Since the resolution of the GISS model is in large dimensions, using a multivariate regression method, the outputs of the climate parameters are scaled for 87 stations in Tehran and its surrounding areas using MATLAB software (MATLAB 7.5.0.342; R2007b). In this software, the Statistics Toolbox, (multiple linear regression) was used for simulation.

**Figure 1:**
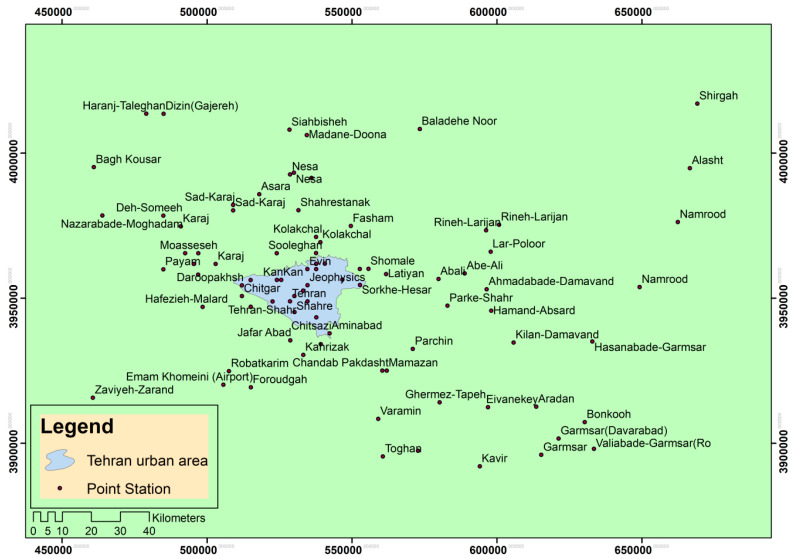
Spatial distribution of stations under study in this research.

In order to show the spatial distribution of the changes in the microclimatic model of Tehran for two basic and future periods, using Arc GIS software and Kriging interpolation method, their maps were produced and presented. Figure 1 shows the distribution of the stations used in the present study.

### 2.1. Effective Temperature

ET is one of the oldest physiologic comfort indices identified to be applicable to Africa [40]. However, for many parts of the world, such as Nedel et al. (2015) [41], for Brazil, Robaa and Abdel Wahab (2018) [42] for Palestine, Wu et al. (2018) [43] for China, this indicator is used, with the results being approved. It is defined as the temperature at which saturated air would make a normal person to wear ordinary indoor clothing. It was originally intended for indoor conditions in industry and mines and not for open air conditions but has later gained wider applications in comfort and climatologic analyze [44]. An approximation of the ET is given in either of these forms [45]:

*T_eff_* = *t* − 0.4(*t* − 10)(1 − *H_rh_*/100) (1)

*T_eff_* = 0.4(*T_dry_* + *T_wet_*) + 4.8 (2)

where *T_eff_*: effective temperature; *t*: air temperature; *H_rh_*: is relative humidity; *T_dry_* and *T_wet_*_:_ are dry and wet bulb temperatures, respectively.

Uncomfortable situations due to cold stress occur at ET ≤ 18.9 °C, and that due to heat stress at >25.6 °C [40]. Observations by Gregorczuk and Cena (1967) [44] suggested that ET over latitudes 10–30°N was highest in the world in July and that the spatial distributions of ET values are similar to that of the air temperature, sometimes modified by high relative humidity.

It is worth noting that the process of calculating the effective temperature index for two hours 3 and 15 GMT was done to compare the results in these two hours.

### 2.2. Introducing the Method of Projecting and Downscaling of Climate Variables

First, a temporal series of the data for dry and wet temperature in monthly-scale data for the years 1971 to 2010 was designed to simulate and exponential downscaling of these components for future decades were provided. Then, from the NASA Meteorological Center (thredds-ncss-grid-CMIP5), the data related to the diagnostic components of the general GISS model for the range of 30 to 40 degrees north and 50 to 55 degrees east in the form of 18 knots 2.5 × 2.5 degrees was received. The predictive components of the GISS model include 81 general atmospheric components (Table 1). 

In order to diagnose and exponential downscale of wet and dry temperature data of the study area, the best correlation between circulating components for the base period was used. The selected period for this purpose was from 1971 to 2005, and then, for training and testing, the baseline period was divided into two periods from 1971 to 2000 and 2001 to 2005. It needs to be explained that the output of the general circulation model of the atmosphere is based on two scenarios RCP4.5 and RCP8.5 from 2006 onwards. Thus, in a separate process, we also used actual data from stations for 2006 to 2014 to validate the outcomes of the two scenarios mentioned.

Finally, according to the parametric correlation method (Pearson), the extracted relations were evaluated and then, based on the coefficients of determination and testing the residuals’ independence, based on Durbin-Watson method and the other statistical methods shown in Table 2, the results of the modeling were verified.

Therefore, based on the least error in modeling, it was determined that the best relation for diagnosing and exponential downscaling of climate variables of wet and dry temperatures were obtained with three minimum and maximum of temperature and specific humidity components. These variables in Table 1 include rows 18, 19, and 60, which are also highlighted in italic font. In order to complete the above, it should be noted that each of the components in Table 1, were used in temporal scales of current and future period (up to the 2050s) and based on two scenarios RCP4.5 and RCP8.5.

### 2.3. Introducing Statistical Methods to Calibrate and Validate the Climate Model

In the present study, in order to calibrate the results of projecting and downscaling of climate variables, the Equation (3) was used. Since in all years the average annual standard deviations of all variables are lower than the standard deviation of the observe period and itis expected that climatic limit values increase in the future, in order to resolve this shortcoming using Equation (3) while maintaining the means, standard deviation of these variables are increased in the basic period with the ratio of the standard deviation of observed data to the simulated data by the model of the past period.

(3) $$F_{fut}=\left(\frac{Sim_{i}-Sim_{ave}}{STD_{sim}}\times STD_{obs}\right)+Obs_{avg}$$

In Equation (1), the *F_fut_* component contains calibrated values, and *Sim_i_* is introduced as the simulated value of *i*-th and *sim_ave_* and *STD_sim_* are the mean and standard deviation of simulated values, respectively. Moreover, *STD_obs_* and *Obs_ave_* are the mean and standard deviations of observational values, respectively.

After calibrating the simulated data, in order to evaluate their fit with real data, the Nash-Sutcliffe test was used. The Nash–Sutcliffe Goodness of Fit statistics [46] is computed as follows:

(4) $$NS=1.0-\sum_{n=1}^{ntot}{\left(OBS_{n}-SIM_{n}\right)}^{2}/\sum_{n=1}^{ntot}{\left(OBS_{n}-MN\right)}^{2}$$

where $OBS_{n}$ is observed climatic parameters (wet and dry temperatures), and $SIM_{n}$ is simulated climatic parameters. 

NS can range from −∞ to 1. An efficiency of 1 (*NS* = 1) corresponds to a perfect match of simulated values to the observed data. An efficiency of 0 (*NS* = 0) demonstrates that the model predictions are as accurate as the mean of the observed data. In essence, closer the efficiency of the model is to 1, the more accurate is the model [47]. After calibrating the simulated climatic data with the stations real data, in the next step, using the statistical methods, Root Mean Square Error (RMSE), Bias Method (MBE) and rate of R-Square, the validity of the simulated data calibrated with the stations real data was evaluated and the results are presented in Table 2, Table 3, Table 4 and Table 5. The least rate of RMSE is zero. The great deals of MAE would be an indicator of the worst status of similar action, while the RMSE indicates how much maximum and minimum the estimation is, compared to the observed data. In the best status of the numeric rate, RMSE would be close to zero. MBE indicates how different the estimated and the observed rates are. If the rate equals zero, it indicates the deals are good examples of simulation, but in most cases the difference is in a way that the further it is from zero, the weaker simulation becomes. R-Square is in fact the so-called determination index, which indicates the correlation ratio of the two variables, and if multiplied by 100, it can show the impact ratio percent [48].

### 2.4. Shannon Entropy Indicator 

One of the objectives of this paper is to investigate the irregularity or balance of ET values under real conditions of observational data and comparison with simulated data of next decades for Tehran. Therefore, one of the strategies for achieving this goal is to use the Shannon entropy method. 

In information theory, Shannon’s entropy is regarded as a measure of variability or randomness in the data, which is analogous to the lack of information about the system [49,50]. Shannon’s entropy (*H*) of a random variable (such as time-series data) is calculated as [51]:

(5) $$H=-\sum_{i=1}^{B}P_{i}log_{2}(P_{i})$$

where, *B* is the set of measurements and *P_i_* denotes the probability of outcome as *i* varies from 1 to *B*. Equation (5) suggests that the value of entropy varies according to the distribution of *P_i_*’s associated with the set *B* chosen to represent the random variable. This implies that by increasing the number of constraints, or by specifying more information about the random variable, the range of entropy decreases. Therefore, process components that add information to the system reduce Shannon’s entropy and are able to explain the variability in the data series [25,52,53,54]. Finally, entropy maps were produced based on the results of normalized entropy calculations. It is necessary to explain that *H_n_* is the normalized entropy because the relative frequency entropy was calculated Equation (6)

(6) $$H_{n}=\frac{-\sum_{i=1}^{B}P_{i}log_{2}(P_{i})}{log_{2}\left(B\right)}$$

**Figure 2:**
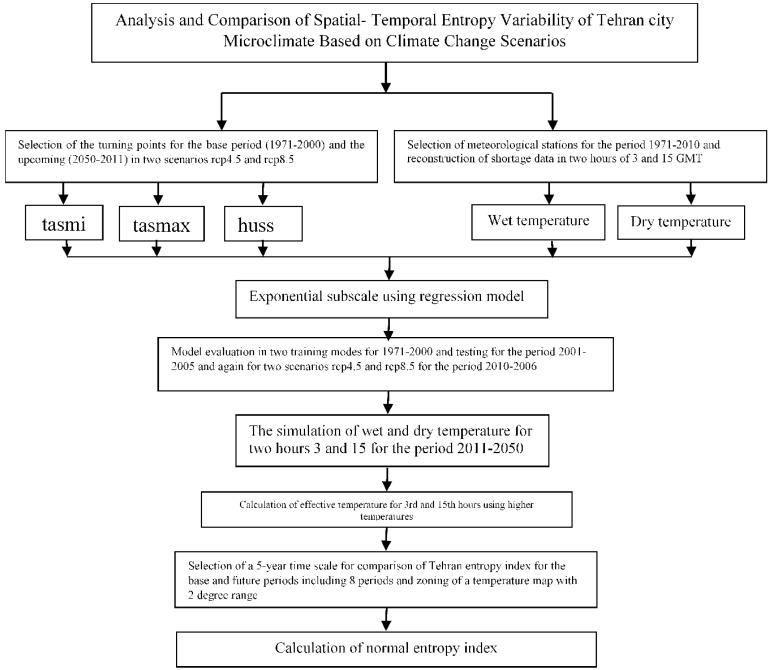
Flowchart of various stages involved in research study.

The steps for this section were that after the exponential downscaling of the wet and dry temperatures, the effective temperature was first calculated for two hours of 3:00 and 15:00. Then, for the region of Tehran, the effective temperatures in a 50 × 50 matrix with a horizontal resolution of 1118 m and a vertical of 800, were simulated for all months from 1971 to 2050. Then, to calculate the frequency of effective temperature, we set a temperature range of 2 degrees, so that for every decade, the percentage of relative frequency was calculated for each pixel. Finally, due to the preparation of relative frequency, the temporal entropy was obtained using Equation (6). Figure 2 show various stages involved in research study.

## 3. Research Findings

### 3.1. Validating Climate Modeling Results

In order to verify the exponential downscaling accuracy, the temperature data of the base period was divided into three periods of time, in which the period 1971 to 2000 was selected as the training model test period and periods 2001–2005 and 2006–2014 as the model test periods. 

However, it should be noted that the last period is a test in two time scenarios since from the beginning of this period, the output of scenarios RCP 4.5 and RCP8.5 at macro-level is provided for future periods. In order to achieve this, in addition to determining the error rate (RMSE), deviation from the fit line (BIAS) and coefficient of determination (R^2^), the Nash–Sutcliff criterion (NS) was used to evaluate the accuracy of the model. This criterion ranges from negative infinite to one, and if it reaches one, there will be a perfect fit between simulated and observational data. According to Table 2, it is determined that according to the Nash–Sutcliff criterion, the model has a high efficiency for the exponential downscaling for future periods. As can be seen, its average for dry temperatures at 3:00 for the training period (2000–1791) is 0.941. The maximum value was 0.963 and the minimum was 0.872. Additionally, the values for this test statistic for the experiment period (2001–2005) include an average of 0.939, with a maximum of 0.957 and a minimum of 0.908. On the other hand, the Nash–Sutcliffe test outputs for the RCP4.5 and RCP8.5 scenario data for the period from 2006 to 2014 indicate the high output of this test, which confirmed the efficiency of climate modeling. The dry temperature test of 15:00 also indicates a high performance of the exponential downscaling model (Table 3).

The wet temperature test for 3:00 and 15:00 GMT also shows that for the training period (1971–2000), the average output of the Nash-Sutcliffe test was 0.935 and 0.928, respectively, with a maximum of 0.951 and 0.947 for these two hours, respectively, and its minimum values are 0.892 and 0.888. However, for 3:00 and 15:00 GMT, the average Nash–Sutcliffe test for the trial period (2001–2005), for 3:00, was 0.933, and for 15:00, was 0.924, with a maximum of 0.959 and 0.944 for these two hours. On the other hand, the minimum test value for 3:00 is 0.842 and for 15:00 it is 0.759. The comparison of the two scenarios 4.5 and 8.5 during the period 2006–2014 suggests that, although in the 8.5 scenario, the performance of the model is weaker than 4.5, the criterion is to be precise in terms of exponential downscaling and diagnosis. In Table 4 and Table 5, the results of all tests for diagnostic validation and the exponential downscaling of climate modeling for two climatic components of wet and dry temperatures at 3:00 and 15:00 GMT have been provided. Outputs confirm the appropriate validity of the proposed climate modeling in the present study. In order to summarize the results of this section, they are presented as tables.

### 3.2. Zoning of Effective Temperature Index for Different Study Periods

**Figure 3:**
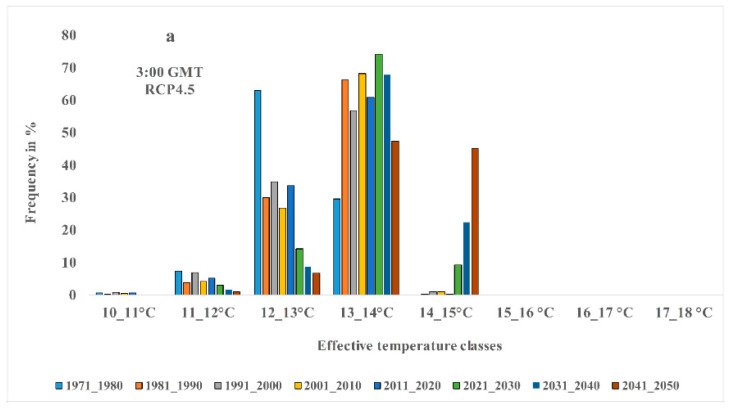

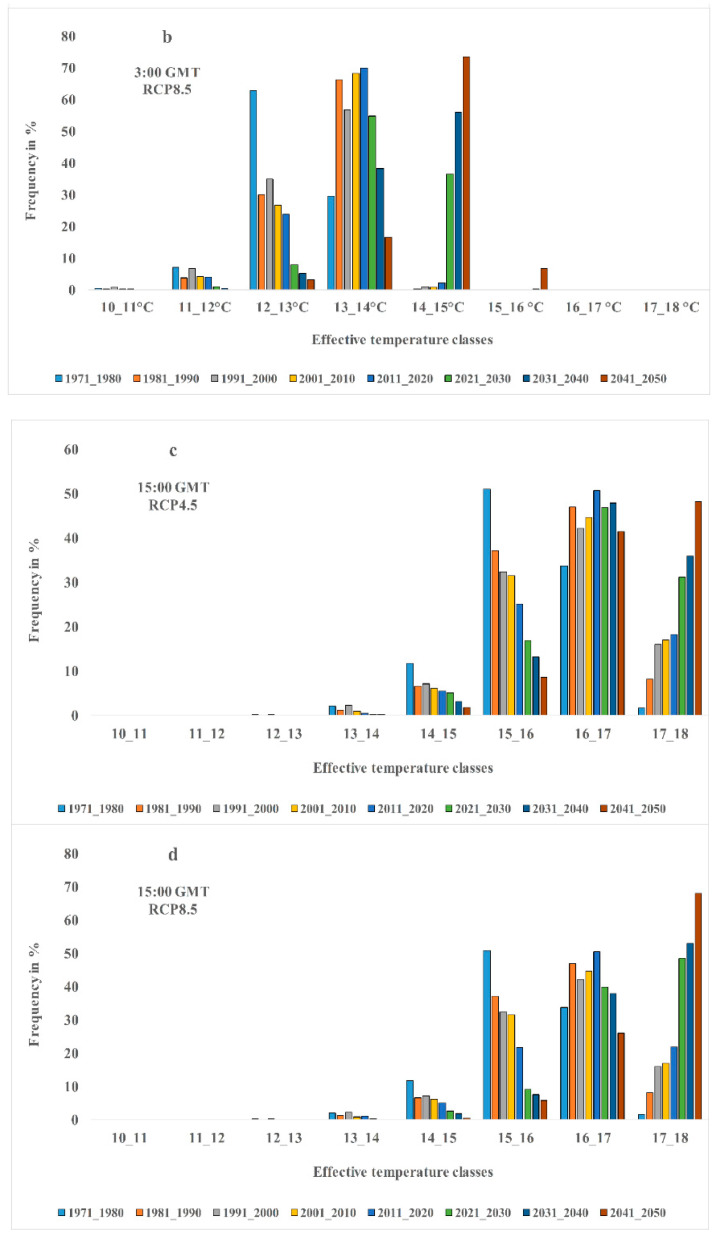
Comparison of the effective temperature of different classes for different hours and scenarios for the past to future decades.

In this part of the study, eight decades of study have been considered in order to study the effective temperature changes in Tehran. The first period covers 1970 to 1980, and the last period includes 2040–2050. Thus, for both 3:00 and 15:00 GMT, and two RCP4.5 and RCP8.5 scenarios, temporal-spatial variations of the effective temperature have been monitored and projected. It should be explained that nine classes were considered for the effective temperature event. The first class has a temperature range of 10 to 11 degrees Celsius, and the last class is from 18 to 19 degrees Celsius. Therefore, temporal and spatial variations of different effective temperature classes for different periods are presented as maps. In addition in Figure 3, the percentage of effective temperature area is given in different classes. Initially, outputs are analyzed for both scenarios and at 3:00 GMT. As can be seen from Figure 3, for the RCP4.5 scenario, the effective temperature values for classes greater than 15 °C and RCP8.5 for temperature classes greater than 16 °C are not considered. However it is interesting to note that for the first class, the temperature of 10 to 11 degrees Celsius, there will be no events for the next decade, and no area in Tehran will experience this temperature threshold. This can mean higher temperature thresholds than the lower temperatures for decades to come. 

**Figure 4:**
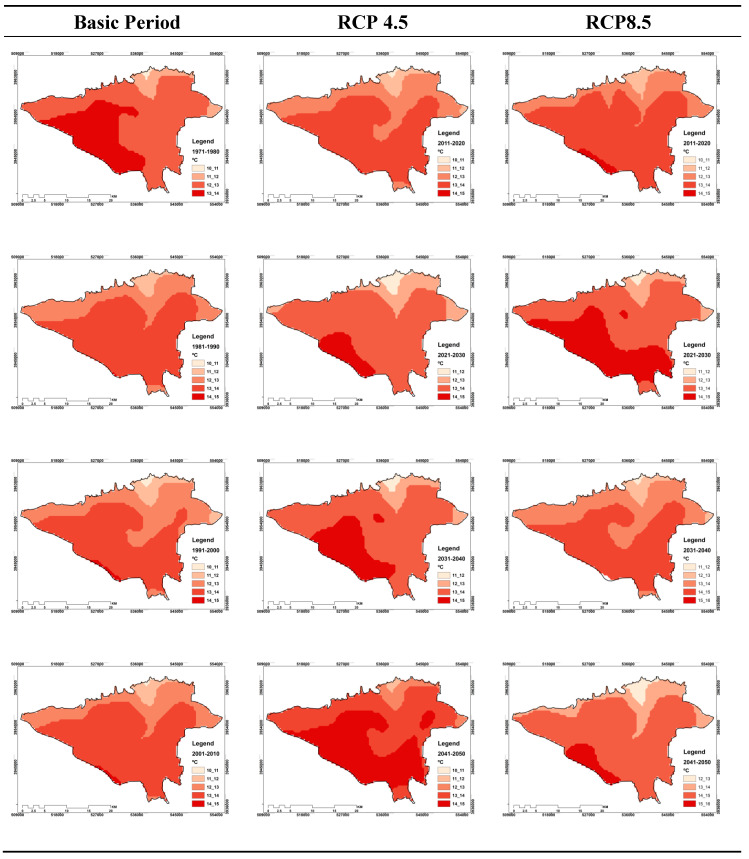
Monitoring and projecting of temporal–spatial distribution of different classes of effective temperature indicator at 03:00 GMT in Tehran.

On the other hand, outputs for this hour show that the maximum area of Tehran in different times covers temperatures of 13 to 14 degrees Celsius, which is significant for both scenarios. However, based on the RCP4.5 scenario, most of the zones that experience 14 to 15 degrees temperatures are in the future decades, especially in 2030 decade. Thus, for this class in the first two decades, no event experience is seen. However, for the RCP8.5 scenario, the situation is different. Because the occurrence of temperature classes of 15 °C to 16 °C is seen for the first time since the 2030s onward. The claim is that the temperature threshold is 15 to 16 degrees Celsius, respectively, with an area of 0.25 and 6.79 percent for the two decades of 2030 and 2040. Note that, as can be seen in Figure 4, the maximum values or greater values of the effective temperature are for the southern half of Tehran, and the minimum are for the northern half. These conditions are for both present and future and both scenarios as well.

The findings for the 15:00 GMT show significant differences compared to the 03:00 GMT. For this hour, in both scenarios, there is no experience for two primary classes, which include temperature thresholds of 10 to 11 and 11 to 12 degrees Celsius. On the other hand, the results of Figure 4 show that the maximum effective temperature range based on different periods of study is related to the temperature class of 16 to 17 degrees Celsius, which is true for both scenarios. 

**Figure 5:**
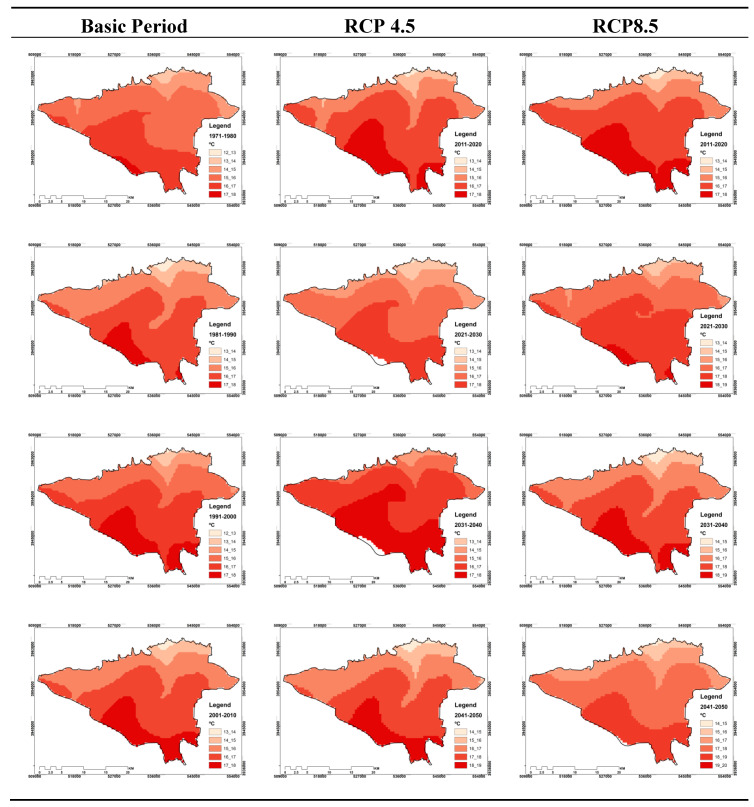
Monitoring and projecting of temporal-spatial distribution of different classes of effective temperature indicator at 15:00 GMT in Tehran.

The temperature range from 17 to 18 degrees Celsius is the last temperature class in which the zones of Tehran experience or will experience its temperature in different periods. While it is observed that, based on global warming, the percentage of the area in Tehran experiencing this temperature class will be larger in future decades than in the past decades. Additionally, in comparison of RCP4.5 with RCP8.5, more zones of Tehran based on the RCP8.5 scenario include this temperature class. For example, for the 2020–2030 decade, the area of this category was 31.15% for the RCP4.5 scenario and 48.56% for RCP8.5, which were 35.85 and 52.96 percent in the 2030-2040 for RCP4.5 and RCP8.5, respectively. Finally, for the 2040–2050 decade, the area covered by RCP4.5 was 48.2% and the area covered by RCP8.5, was 67.87%. However, in terms of spatial distribution and its changes in time, it is also observed that the south of Tehran has the highest effective temperature values, which can be seen in the same scenario for both current and future periods based on the two scenarios (Figure 5).

### 3.3. Spatial–Temporal Analysis of Shannon’s Entropy Values from the Effective Temperature Index

As previously explained, the effective temperature index values for the two hours of 3:00 and 15:00 GMT and for the two basic and future periods are calculated and modeled. Then, on the basis of spatial–temporal distribution, different values of the effective temperature occurrence at the study area level, Shannon entropy values were calculated and its distribution map was generated. Therefore, at this stage, the maps shown are merely zoning the Shannon entropy temperature. However, in order to complete the above discussion, it is worth noting that in order to compare the Shannon entropy variations of the effective temperature in the time frame, the maps of the study periods were produced for an average of eight periods. 

The first period consisted of the average 1971–1980 and the last period, which included the average years from 2040 to 2050. In this study, for the purpose of proper comparison between different maps, the smallest entropy incidence class was considered 0.9 and its maximum was 0.99. Therefore, zones that tend to be high in entropy are accompanied by disorder, more climatic and microclimatic disturbances, and the zones that exhibit minimal entropy are at least influenced by climatic anthropogenic. The results of 3:00 GMT are presented below:

#### 3.3.1. Shannon Entropy Effective Temperature Indicator for 3:00 GMT

**Figure 6:**
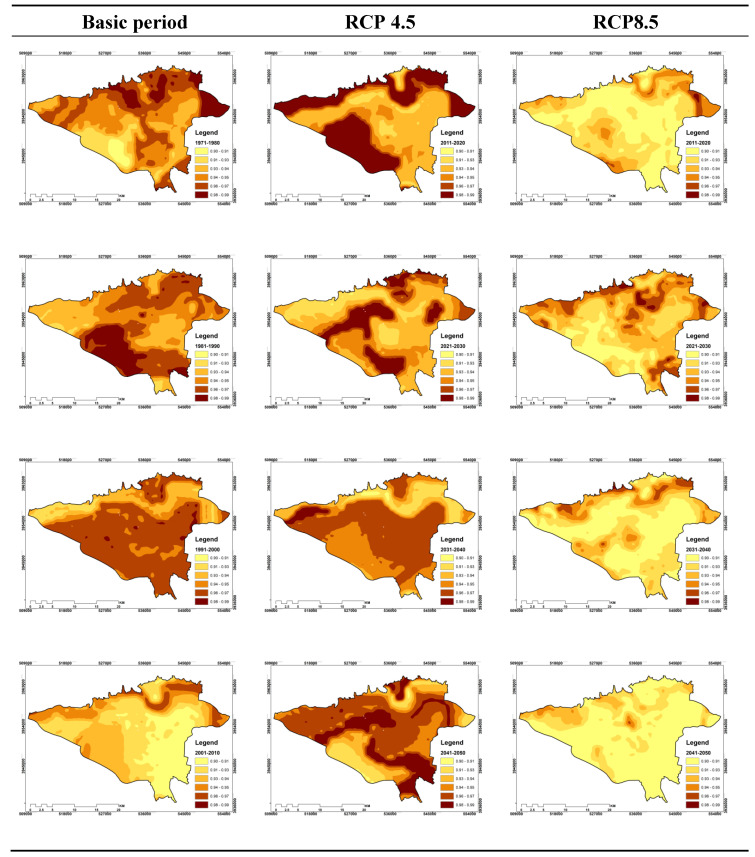
Spatial–temporal distribution of Shannon entropy values of effective temperature for 3:00 GMT for the decades under study.

For this hour, the results of the RCP4.5 are presented from the mean of the first decade to the last decade as shown in Figure 6. Based on this Figure, it can be seen that the spatial–temporal distribution of Shannon entropy of the effective temperature does not follow a particular pattern for this hour and in different periods. 

For example, in the first decade, its minimum value is related to a zone in the southwest of the study area, while in the subsequent period, this region has the maximum amount of entropy index. However, in the third decade or the same decade from 1991 to 2000, the minimum incident was transferred to the northern bar. However, in 2001–2010, no track of the class 0.98 to 0.99 class are seen, and the majority of the area is in the range of 0.90 to 0.95. 

In addition, this entropy and the lack of a regular process in the dispersion of spatial variations of Shannon Entropy for the upcoming periods are also seen. Because in the period of the zone where the entropy has the maximum amount it has been miniaturized in the next period or for each period the maximum and minimum area of the Shannon entropy zone is located in a different range that cannot be dispersed. Therefore, no comprehensive result can be achieved from such entropies. At this stage, the incremental and decreasing area of Shannon entropy was calculated for the next period compared to the previous period and finally the average of these changes was calculated. Interestingly, the overall average of the areas where the amount of Shannon entropy is incremental to them is 51.7%, and this average is 63% for the overall reduction of the overall period.

Based on the scenario of RCP8.5, spatial–temporal variations of the Shannon entropy for effective temperature index are more orderly than RCP4.5 results (Figure 6). Altogether, for the first observation period, the maximum entropies were related to the northern zones, which during the second period of observation, this maximum were transmitted to the southern regions of the area. From the decade 2000 to 2010, the minimum entropy values are again transferred to the northern regions, and from this time until the end of the 1990s, the decreasing trend of entropy values for the study area is considered. 

However, in the 2021–2030, the Shannon entropy values increased in comparison with the previous periods, but again they showed a decreasing trend for the last two decades. In sum, it can be admitted that the decreasing trend of Shannon entropy values for the effective temperature index at 3:00 has been more than the increasing trend for the decades under study. 

The outcomes of this section showed that the total mean of the Shannon entropy for the entire study period is 36.51%, which is 63.49% for the reduction values. Therefore, it can be concluded that the decreasing trend of Shannon entropy values of the effective global heating temperature is decreasing for Tehran and the suburbs at this hour of the day.

#### 3.3.2. Shannon Entropy of Effective Temperature Indicator for 15:00 GMT

For 15:00 GMT, the results are more interesting than 3:00. So, for the RCP4.5 scenario, with a general look, it can be seen that from the first decade to the last decade, the time variation of the Shannon entropy incident is decreasing. 

**Figure 7:**
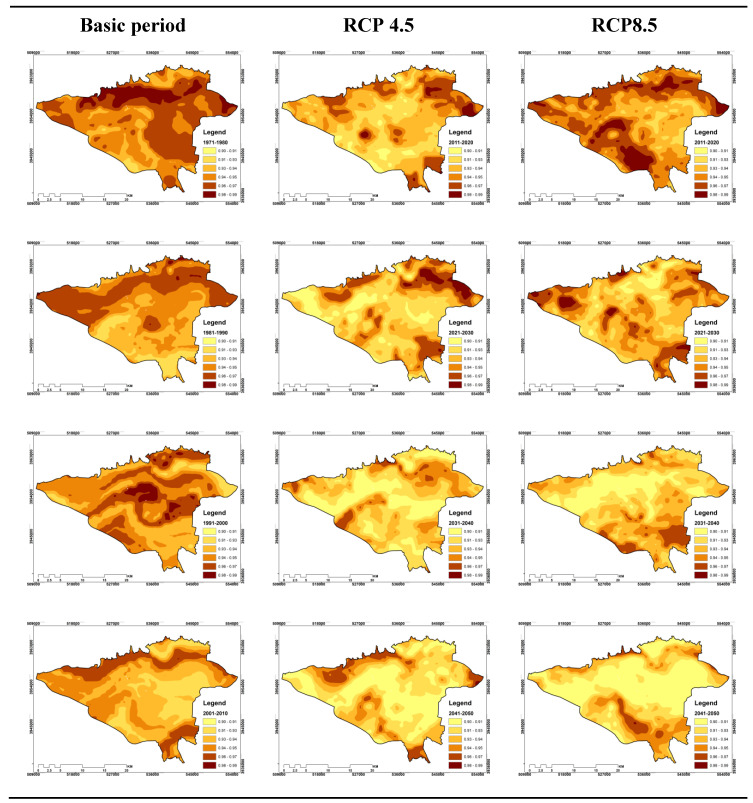
Spatial–temporal distribution of Shannon entropy values of effective temperature for 15:00 GMT for the decades under study.

On the whole, based on Figure 7, for the northern observation period, the region exhibits the greatest disturbances or the highest entropy values. However, the same is true for decades to come. So that the northern parts and northern borders of the region continue to appear as the most entropic spots in comparison to other areas. Estimates from the changes in the area of increase and decrease of Shannon entropy for different periods showed that its average increment for the whole decade of study was 39.56%. According to global warming, an average of 39.56% of the area of the study area experienced an increasing increase in Shannon entropy over time. While 60.43% of the area of Tehran and the suburbs, the variability of Shannon entropy for them shows a decrease (Figure 7). However the findings for RCP8.5 are similar to RCP4.5. Because at a glance, the temporal and spatial variations of the Shannon entropy of the effective temperature at 15:00, from the first decade to the last decade, are decreasing. Thus, on average, 40.74% of the Tehran area experienced increasing amounts of Shannon entropy coefficient, and on the other hand, 59.25% of its area is considered decreasing values (Figure 7). Although it is difficult to analyze which region has experienced the maximum entropies in the whole study period, it can be argued that most northern band and parts of the west and the north east have the smallest amount of Shannon entropy (Figure 7).

## 4. Discussion 

Tehran, in the south of the Alborz Mountains, today faces three types of climate-related hazards from geography, the climate risk resulting from air sustainability and global warming climate risks. The massive concentration of population in this geographical area has increased the impact of these hazards. The importance and necessity of this research has been that Tehran as a political center of Iran with a population of tens of millions today has more than 200 days of atmospheric pollution in combination with the occurrence and intensification of heat islands and increasing thermal stress. Tehran’s encirclement in an arched space reduces the purification property of the western and southeast winds, and therefore, in most cases, the air stays idle. As a result, this situation can have an impact on the warming of the heat islands and thermal stresses, especially during the warm period of the year and affect the health of the community. Therefore, in this research, the effect of global warming on the changes in the bioclimatic component of ET was investigated for two current and future periods, based on different scenarios. In the next step, the spatial–temporal variability of Shannon entropy in the effective temperature of Tehran city was studied for different periods in order to determine the effect of anthropogenic anomalies on the effective temperature index. The results of this study showed that the effective temperature index has been rising on base of global warming for the upcoming decades. These incremental values are visible for both RCP4.5 and RCP8.5 scenarios. It can be seen that the magnitude for the RCP8.5 scenario is more than the other scenario. However, one of the main index for calculating the bioclimatic condition is index of ET. Therefore, population growth and urban buildings, increasing greenhouse gases, land use change, etc. have been the most important factors influencing climate change and temperature rise in Tehran. 

In many other studies of Iran, it is clear that with regard to global warming, the process of temperature change is incremental; this is a credit to the findings of this study. One of these findings was from a study by Roshan and Negahban (2015) [55] for the southwest of Iran. They simulated temperature changes from 2020 to 2060 using the output of GCMs (HadCM3) based on LARS-WG software. The results of the study indicated an increase in temperature from 0.32 °C to 0.51 °C compared with temperatures in the 1988 to 2010 base period. Ghanghermeh et al. (2015) [56] divided Iran into seven clusters according to the statistical period of 1948–2010. Their results showed that there was an increasing trend in temperature for all clusters. The results of Tiseuil et al. (2012) [57] for northwest regions of Iran showed that by 2100 the temperature will increase by 1.5 °C for these areas. In many other studies in other parts of the world, the outputs of GCMs relate global warming with temperature increase [58,59,60,61,62].

In this study, the temporal–spatial variability of Shannon entropy was evaluated for two hours of the day, including 3:00 and 15:00 GMT. Overall, the findings showed that, for 3:00, spatial and temporal variability during the study periods did not follow a systematic trend, which was more significant for the RCP4.5 scenario. However, at 15:00, the process of variability is more regular. According to the entropy findings, the increase of the zones whose entropy coefficients are less than the previous periods is increased. This claim can be seen in the following outputs. In such a way that the amount of increase and decremented amount of Shannon entropy for the next period was calculated, and finally the average of these changes was calculated. Interestingly, the overall average of the areas where Shannon entropy has been increasing for RCP4.5 at 3:00 is 51.7%, and this average for the reduction of the overall period is 63%, but these incremental and decreasing changes for The RCP8.5 scenario includes 36.51% and 63.49%, respectively. However, at 15:00, these patterns are also observed. According to the RCP4.5 scenario for this hour, the average total incremental variation of the entropy coefficient was 39.56% and the decrease was 39.56% of the study area, and for the RCP8.5 scenario, on average, 40.74% of the Tehran area increased the Shannon entropy coefficient and for 59.25% of its area, there are decreasing values. 

## 5. Conclusions

What does this Shannon entropy coefficient variation model mean in terms of effective temperature from the point of view of urban climatology? However, as a new step in the research about climate change in Tehran city due to global warming [22,23], the temperature difference between the different areas of the study area has declined, which is believed to reduce the abnormalities and more orderly between the data spatially and over time. It is observed that the lowest values of the Shannon entropy occurred in the last two decades, from 2030 to 2040, and the other 2040–2050. Because, based on global warming, dominant areas have increased temperature, and the difference in temperature is reduced daily and the temperature difference between the zones of different areas is less. 

Therefore, this subject is considered order from the point of view of entropy, but the urban climate perspective does not have a positive view of this process. However, the presence of temperature differences can lead to a difference in local pressure and wind power production. Therefore, in the urban area, the local wind flow can reduce the thermal stress to a certain extent and affect the flow and displacement of stagnant layers of the air and also reduce the city’s thermal budget. While the uniformity of temperature between different regions causes weakness in the production of local winds, local wind currents cannot be effective as a mechanism for the displacement and air-tightness of the air. Thus, in the future decades, more people are expected to endanger their health in terms of thermal stress. Therefore, the management of the city of Tehran should act in such a way that land use does not lead to the loss of agricultural land and its suburban lands. Considering the creation of urban green spaces, green roofs, urban design, artificial lakes and artificial zones can also contribute somewhat to excessive load of temperature and to reduce thermal stress.

## Figures and Tables

**Table 1:** 81 diagnostic components of the GISS general circulation model.

Number of Row	Circulate Components	Number of Row	Circulate Components
1	zg = Geopotential Height	42	ra = Carbon Mass Flux into Atmosphere due to Autotrophic (Plant) Respiration on Land
2	wap = omega (=dp/dt)	43	psl = Sea Level Pressure
3	vo = Sea Water Y Velocity	44	ps = Surface Air Pressure
4	vas = Northward Near-Surface Wind	45	prw = Water Vapor Path
5	va = Northward Wind	46	prveg = Precipitation onto Canopy
6	uo = Sea Water X Velocity	47	prsn = Snowfall Flux
7	uas = Eastward Near-Surface Wind	48	prc = Convective Precipitation
8	ua = Eastward Wind	49	pr = Precipitation
9	tsl = Temperature of Soil	50	npp = Carbon Mass Flux out of Atmosphere due to Net Primary Production on Land
10	ts = Surface Temperature	51	nep = Net Carbon Mass Flux out of Atmosphere due to Net Ecosystem Productivity on Land.
11	transiy = Y-Component of Sea Ice Mass Transport	52	mrsos = Moisture in Upper Portion of Soil Column
12	transix = X-Component of Sea Ice Mass Transport	53	mrso = Total Soil Moisture Content
13	tran = Transpiration	54	mrros = Surface Runoff
14	tos = Sea Surface Temperature	55	mrso = Total Soil Moisture Content
15	thetao = Sea Water Potential Temperature	56	mrro = Total Runoff
16	tauv = Surface Downward Northward Wind Stress	57	mrlsl = Water Content of Soil Layer
17	tauu = Surface Downward Eastward Wind Stress	58	mrfso = Soil Frozen Water Content
*18*	*tasmin = Daily Minimum Near-Surface Air Temperature*	59	mc = Convective Mass Flux
*19*	*tasmax = Daily Maximum Near-Surface Air Temperature*	60	*huss = Near-Surface Specific Humidity*
20	tas = Near-Surface Air Temperature	61	hus = Specific Humidity
21	ta = Air Temperature	62	hurs = Near-Surface Relative Humidity
22	sos = Sea Surface Salinity	63	hur = Relative Humidity
23	so = Sea Water Salinity	64	hfss = Surface Upward Sensible Heat Flux
24	snw = Surface Snow Amount	65	hfls = Surface Upward Latent Heat Flux
25	snm = Surface Snow Melt	66	gpp = Carbon Mass Flux out of Atmosphere due to Gross Primary Production on Land
26	snd = Snow Depth	67	evspsblveg = Evaporation from Canopy
27	snc = Snow Area Fraction	68	evspsblsoi = Water Evaporation from Soil
28	sit = Sea Ice Thickness	69	evspsbl = Evaporation
29	sic = Sea Ice Area Fraction	70	evap = Water Evaporation Flux from Sea Ice
30	sfcWind = Near-Surface Wind Speed	71	clwvi = Condensed Water Path
31	sci = Fraction of Time Shallow Convection Occurs	72	clw = Mass Fraction of Cloud Liquid Water
32	sbl = Surface Snow and Ice Sublimation Flux	73	clt = Total Cloud Fraction
33	rtmt = Net Downward Flux at Top of Model	74	clivi = Ice Water Path
34	rsutcs = TOA Outgoing Clear-Sky Shortwave Radiation	75	cli = Mass Fraction of Cloud Ice
35	rsut = TOA Outgoing Shortwave Radiation	76	cl = Cloud Area Fraction
36	rsuscs = Surface Upwelling Clear-Sky Shortwave Radiation	77	ci = Fraction of Time Convection Occurs
37	rsus = Surface Upwelling Shortwave Radiation	78	cct = Air Pressure at Convective Cloud Top
38	rlus = Surface Upwelling Longwave Radiation	79	ccb = Air Pressure at Convective Cloud Base
39	rldscs = Surface Downwelling Clear-Sky Longwave Radiation	80	cSoil = Carbon Mass in Soil Pool
40	rlds = Surface Downwelling Longwave Radiation	81	baresoilFrac = Bare Soil Fraction
41	rh = Carbon Mass Flux into Atmosphere due to Heterotrophic Respiration on Land	-	-

**Table 2:** Testing the exponential downscaling of dry temperature model at 3:00 GMT.

Dry Temp at 3:00 GMT	Verification	Period	Mean	Max	Min
**RMSE**	Training	1971–2000	2.049	2.899	1.680
	Testing	2001–2010	2.026	2.597	1.557
	Testing rcp4.5	2011–2014	2.067	2.689	1.646
	Testing rcp8.5	2011–2014	2.094	2.982	1.787
**R^2^**	Training	1971–2000	0.941	0.963	0.872
	Testing	2001–2010	0.943	0.958	0.914
	Testing rcp4.5	2011–2014	0.948	0.962	0.917
	Testing rcp8.5	2011–2014	0.941	0.949	0.905
**BIAS**	Training	1971–2000	0.454	7.896	−23.570
	Testing	2001–2010	0.384	2.571	−12.103
	Testing rcp4.5	2011–2014	0.560	4.003	−6.217
	Testing rcp8.5	2011–2014	0.584	4.237	−4.572
**NS**	Training	1971–2000	0.941	0.963	0.872
	Testing	2001–2010	0.939	0.957	0.908
	Testing rcp4.5	2011–2014	0.938	0.959	0.886
	Testing rcp8.5	2011–2014	0.937	0.948	0.895

**Table 3:** Testing the exponential downscaling of dry temperature model at 15:00 GMT.

Dry Temp at 15:00 GMT	Verification	Period	Mean	Max	Min
**RMSE**	Training	1971–2000	2.326	2.785	1.840
	Testing	2001–2010	2.308	3.148	1.770
	Testing rcp4.5	2011–2014	2.233	2.909	1.884
	Testing rcp8.5	2011-–2014	2.248	2.990	1.934
**R^2^**	Training	1971–2000	0.946	0.962	0.903
	Testing	2001–2010	0.949	0.961	0.910
	Testing rcp4.5	2011–2014	0.956	0.968	0.901
	Testing rcp8.5	2011–2014	0.956	0.964	0.903
**BIAS**	Training	1971–2000	0.436	3.962	0.213
	Testing	2001–2010	0.399	3.204	0.212
	Testing rcp4.5	2011–2014	0.376	3.214	0.199
	Testing rcp8.5	2011–2014	0.368	2.428	0.203
**NS**	Training	1971–2000	0.946	0.962	0.903
	Testing	2001–2010	0.944	0.961	0.896
	Testing rcp4.5	2011–2014	0.953	0.965	0.896
	Testing rcp8.5	2011–2014	0.953	0.963	0.896

**Table 4:** Testing the exponential downscaling of wet temperature model at 3:00 GMT.

Wet Temp at 03:00 GMT	Verification	Period	Mean	Max	Min
**RMSE**	Training	1971–2000	1.726	2.411	1.476
	Testing	2001–2010	1.652	2.592	1.252
	Testing rcp4.5	2011–2014	1.697	2.922	1.482
	Testing rcp8.5	2011–2014	1.796	2.898	1.615
**R^2^**	Training	1971–2000	0.935	0.951	0.892
	Testing	2001–2010	0.940	0.962	0.876
	Testing rcp4.5	2011–2014	0.947	0.955	0.896
	Testing rcp8.5	2011–2014	0.934	0.945	0.893
**BIAS**	Training	1971–2000	−0.690	12.134	−73.193
	Testing	2001–2010	0.595	9.786	−4.044
	Testing rcp4.5	2011–2014	0.558	6.502	−9.196
	Testing rcp8.5	2011–2014	0.619	7.649	−10.444
**NS**	Training	1971–2000	0.935	0.951	0.892
	Testing	2001–2010	0.933	0.959	0.842
	Testing rcp4.5	2011–2014	0.933	0.952	0.762
	Testing rcp8.5	2011–2014	0.926	0.943	0.766

**Table 5:** Testing the exponential downscaling of wet temperature model at 15:00 GMT.

Wet Temp at 15:00 GMT	Verification	Period	Mean	Max	Min
**RMSE**	Training	1971–2000	1.848	2.515	1.522
	Testing	2001–2010	1.808	3.199	1.525
	Testing rcp4.5	2011–2014	2.028	4.813	1.672
	Testing rcp8.5	2011–2014	1.942	4.875	1.579
**R^2^**	Training	1971–2000	0.928	0.947	0.888
	Testing	2001–2010	0.938	0.954	0.862
	Testing rcp4.5	2011–2014	0.946	0.960	0.906
	Testing rcp8.5	2011–2014	0.939	0.952	0.907
**BIAS**	Training	1971–2000	0.480	3.335	0.212
	Testing	2001–2010	0.411	1.965	0.176
	Testing rcp4.5	2011–2014	0.582	3.407	0.253
	Testing rcp8.5	2011–2014	0.532	3.024	0.198
**NS**	Training	1971–2000	0.928	0.947	0.888
	Testing	2001–2010	0.924	0.944	0.759
	Testing rcp4.5	2011–2014	0.905	0.945	0.337
	Testing rcp8.5	2011–2014	0.914	0.943	0.320
